# Extracellular vesicles involved in growth regulation and metabolic modulation in *Haematococcus pluvialis*

**DOI:** 10.1186/s13068-024-02462-z

**Published:** 2024-01-28

**Authors:** Qunju Hu, Zhangli Hu, Xiaojun Yan, Jun Lu, Chaogang Wang

**Affiliations:** 1 Shenzhen Key Laboratory of Marine Bioresource and Eco-Environmental Science, Shenzhen Engineering Laboratory for Marine Algal Biotechnology, Guangdong Provincial Key Laboratory for Plant Epigenetics, College of Life Sciences and Oceanography, Shenzhen University, Shenzhen, 518060 China; 2 College of Marine Science and Technology, Zhejiang Ocean University, Zhoushan, 316022 China; 3 Auckland Bioengineering Institute, University of Auckland, Auckland, 1142 New Zealand

**Keywords:** Extracellular vesicles, *Haematococcus pluvialis*, Functional analysis, microRNA, Metabolite biosynthesis

## Abstract

**Background:**

Microalgae-derived extracellular vesicles (EVs), which transfer their cargos to the extracellular environment to affect recipient cells, play important roles in microalgal growth and environmental adaptation. And, they are also considered as sustainable and renewable bioresources of delivery nanocarrier for bioactive molecules and/or artificial drug molecules. However, their molecular composition and functions remain poorly understood.

**Results:**

In this study, isolation, characterization, and functional verification of *Haematococcus pluvialis*-derived EVs (HpEVs) were performed. The results indicated that HpEVs with typical EV morphology and size were secreted by *H. pluvialis* cells during the whole period of growth and accumulated in the culture medium. Cellular uptake of HpEVs by *H. pluvialis* was confirmed, and their roles in regulation of growth and various physiological processes of the recipient cells were also characterized. The short-term inhibition of HpEV secretion results in the accumulation of functional cellular components of HpEVs, thereby altering the biological response of these cells at the molecular level. Meanwhile, continuously inhibiting the secretion of HpEVs negatively influenced growth, and fatty acid and astaxanthin accumulation in *H. pluvialis*. Small RNA high-throughput sequencing was further performed to determine the miRNA cargoes and compelling details in HpEVs in depth. Comparative analysis revealed commonalities and differences in miRNA species and expression levels in three stages of HpEVs. A total of 163 mature miRNAs were identified with a few unique miRNAs reveal the highest expression levels, and miRNA expression profile of the HpEVs exhibited a clear stage-specific pattern. Moreover, a total of 12 differentially expressed miRNAs were identified and their target genes were classified to cell cycle control, lipid transport and metabolism, secondary metabolites biosynthesis and so on.

**Conclusion:**

It was therefore proposed that cargos of HpEVs, including miRNA constituents, were suggested potential roles in modulate cell physiological state of *H. pluvialis*. To summarize, this work uncovers the intercellular communication and metabolism regulation functions of HpEVs.

**Supplementary Information:**

The online version contains supplementary material available at 10.1186/s13068-024-02462-z.

## Introduction

Extracellular vesicles (EVs) are a heterogeneous group of membranous nanovesicles secreted by all types of cells, acting as the key mediator of many physiological and pathological processes [1–3]. They consist of diverse contents, including ribonucleic acids, proteins, amino acids, lipids, and metabolites, which present the origin and physiological status of their donor cells [2, 4]. Among which, microRNAs (miRNAs), small noncoding RNAs 19–22 nt in length, which can regulate their cognate target genes at the post-transcriptional level, have been considered as key and most well-studied molecules in EVs [5–7]. miRNAs within EVs have been extensively characterized because miRNAs can be selectively sorted into EVs [4, 7–11]. Thus, miRNAs within EVs can be potentially delivered to their recipient cells to affect the stability and expression of specific target genes [7, 8, 10, 12, 13]. The critical role of EVs is to mediate both short- and long-distance cell-to-cell communication in bacteria, fungi, plants, and animals, and cross species or even kingdoms [14–26]. For their innately repletion of bioactive molecules, plant-derived EVs, especially edible fruit- and vegetable-derived EVs, are gaining increasing attention of researchers for their potential health-promoting activities, including anti-inflammatory, antitumor, immunomodulatory, and microbiota modulation activities [13, 17, 22, 26–30]. Furthermore, the unique morphological characteristics and intrinsic capability to vehicle bioactive biomolecules of plant-derived EVs underpin their modulative role in physiological processes as natural nanocarriers, which offer them promising applications for the delivery of EVs’ cargoes and synthetic drugs [31–33]. Indeed, a growing body of studies have explored the potential usage of plant-derived EVs as the next-generation biotherapeutic molecules and drug delivery nanoplatforms, because they possess several advantages, including sustainability, safety, high stability, high biocompatibility, low toxicity, reduced immunogenicity, and amenability for large-scale manufacturing, in comparison with milk-derived exosomes or artificial nanoparticles [3, 22, 23, 28, 30, 32, 34–36].

Microalgae are a sustainable and renewable source of numerous bioactive metabolites with a range of biological activities and industrial applications [37, 38]. For example, pigments, vitamins, antioxidants, and other compounds concentrated in microalgal with various health benefits (e.g., antioxidant, anti-inflammatory, and antibacterial activities) could be exploited as various products’ therapeutic, cosmetic, and cosmeceutical applications [24, 39, 40]. Microalgal-derived EVs are considered to sort complex cellular constituents, including metabolites, proteins, nucleic acids, and other components of the microalgae cells as their cargos, functionalizing them pharmacological functions similar to those of the original microalgae species [30, 32, 33, 36, 39, 41, 42]. Indeed, EVs can be also purified from microalgal cultivation media and involved in transporting both high-value microalgal substances and bioactive molecules into the extracellular environment [24, 25, 39, 42]. In addition, microalgal-derived EVs are lipid-based carriers capable of carrying and delivering synthetic drug molecules [30, 42]. Thus endowing microalgal-derived EVs with application potential in biomedicine field as both biological therapeutic agent and drug delivery natural nanocarriers [30, 39, 40, 42]. Compared with the usage of other sources for EV production, the usage of microalgae as a natural source for EVs offers several advantages, including environmental sustainability, scalability, controllability, cost-effectively, and renewability, which combine to contribute to the suitability of utilizing these microalgal-derived-EVs as cosmeceutical and therapeutical nanocarriers [24, 41, 42]. In recent years, reports have proposed microalgae as a novel, sustainable, and renewable bio-factory of EVs, which are exploited as tailor-made high-value-added bioproducts of microalgae biotechnology for different industrial sectors, including nanomedicine, nutraceuticals, and cosmetics [24, 39–42]. For example, Adamo et al. [24] have developed and patented a platform for the high-yield, high-quality production of microalgal-derived EVs, and named them as “nanoalgosomes” or “algosomes”. In general, the natural and sustainable origin of microalgal-derived EVs grants them a greater societal acceptance as a source for formulation preparations [24, 40, 42].

*Haematococcus pluvialis* (*Chlorophyceae*, *Volvocales*) is a unicellular freshwater microalga distributed in many habitats worldwide and is known so far the only commercial-scale production source of natural astaxanthin [43, 44]. In addition to astaxanthin, metabolites with high commercial value, including lipids, carbohydrates, and proteins, are also accumulated by this microalgal species, which can be utilized for biofuel, biofertilizer, animal feed, and health products’ production among others [44]. Therefore, to produce numerous compounds from the same feedstock of *H. pluvialis* by carrying out biorefining through the integration of diverse production processes within a single production facility is a considering solution in turn to overcome the issue of high cost [44]. Based on this, the potential exploitation of *H. pluvialis*-derived EVs (HpEVs) as a high-value by-product during biotechnological utilization of microalgal biomass is a noteworthy attempt. For this, the fundamental knowledge of physiology, including their morphology and function in microalgal growth and metabolism, must be determined in HpEVs. In this study, we isolated and characterized HpEVs from the cultivation media of *H. pluvialis*, which was performed at different life-cycle stages of *H. pluvialis*. Potential biological functions of the HpEVs on *H. pluvialis* growth and metabolite biosynthesis were investigated. Furthermore, miRNA profiling was performed to characterize the differences of HpEV components during three growth stages. Meanwhile, the effects of both different HpEVs and their secretion inhibition on *H. pluvialis* were investigated at the transcriptional level. This work first evaluated the HpEVs and their possible functions, providing a research foundation for their utilization as a large-scale production source of natural nanocarriers for high-value bioproduct of astaxanthin.

## Results

### Isolation and characterization of HpEVs

**Fig. 1:**
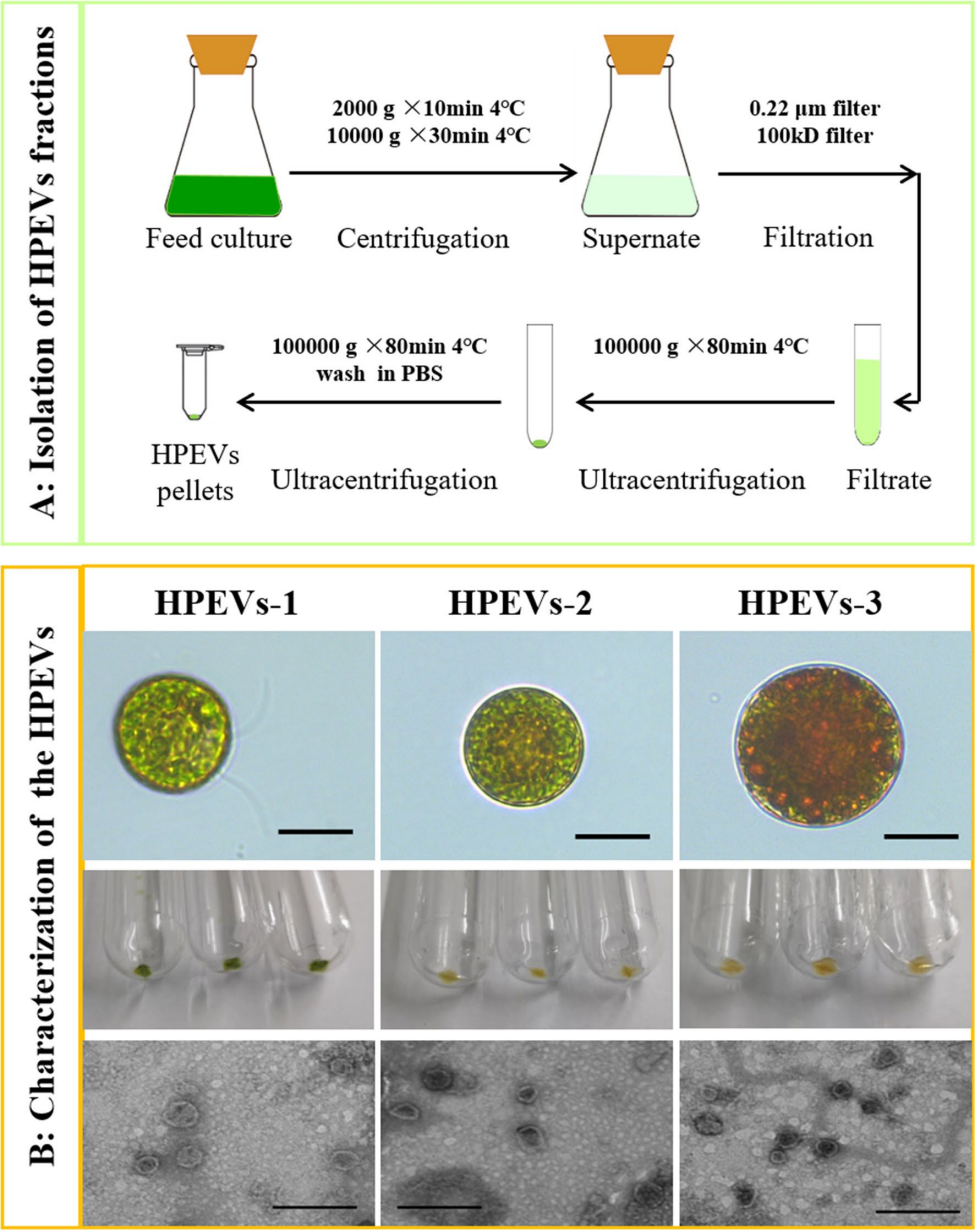
Isolation and characterization of *H. pluvialis* extracellular vesicles (HpEVs). **A** Schematic representation of the method used to isolate and purify HpEVs from *H. pluvialis* cultivation media. **B** Characterization of the isolated HpEVs, successively were microscopic images of *H. pluvialis* cells during different growth stages (scale bar = 20 μm), the isolated HpEVs pellets, and transmission electron microscopy analysis of HpEVs (scale bar = 200 nm). Note that the HpEVs are of similar size and shape across all stages

HpEVs were isolated from the culture media of *H. pluvialis* by differential centrifugation and filtration steps (Fig. 1A). The HpEVs **derived from three stages of *H. pluvialis*, which defined as green vegetative motile stage, green nonmotile stage, and red nonmotile cyst stage of *H. pluvialis* life cycle, were analyzed using a nanoparticle size analyzer and TEM, showing that the HpEVs were spherical or quasi-spherical bilayer-enclosed nanovesicles with diameters of 50–200 nm (Fig. 1B). These negatively stained HpEVs were cup-shaped objects formed by a low-density outer layer and a high-density inner compartment. No apoptotic bodies, cellular debris, and protein aggregates were detected in HpEVs. As shown in Table 1, the particle diameters of HpEVs-1, HpEVs-2, and HpEVs-3 were 89.1 ± 55.7 nm, 112.2 ± 36.2 nm, and 118.2 ± 1.1 nm, respectively. Therefore, no obvious differences in structure and size were observed among the HpEVs derived from three growth stages. Protein concentration of HpEVs-3 was 323.02 ± 2.02 μg mL^−1^, which was significantly higher than that of HpEVs-2 and HpEVs-1. HpEVs-2 also showed higher level protein than that of HpEVs-1 (Table 1). Hence, it might be indicated that the amount of HpEVs increase with *H. pluvialis* stages change from the green vegetative stage to the red cyst stage. These results showed that the *H. pluvialis* cells could produce and release EVs into the culture media.

**Table 1:** Particle size and protein concentration of the different HpEVs

Treatments	Diameter (nm)	Protein concentration (μg mL^−1^)
HpEVs-1	89.1 ± 55.7	69.24 ± 2.02^c^
HpEVs-2	112.2 ± 36.2	75.70 ± 5.48^b^
HpEVs-3	79.2 ± 61.1	323.02 ± 2.02^a^

Different superscript lowercase letters on top of the columns indicate significant differences (*p* < 0.05) among the treatments, and any of the same lowercase letters or letter said the difference was not significant (*p* > 0.05). The following tables are the same

### The effects of HpEVs on the growth and metabolite synthesis of *H. pluvialis*

#### Cellular uptake of HpEVs by *H. pluvialis*

**Fig. 2:**
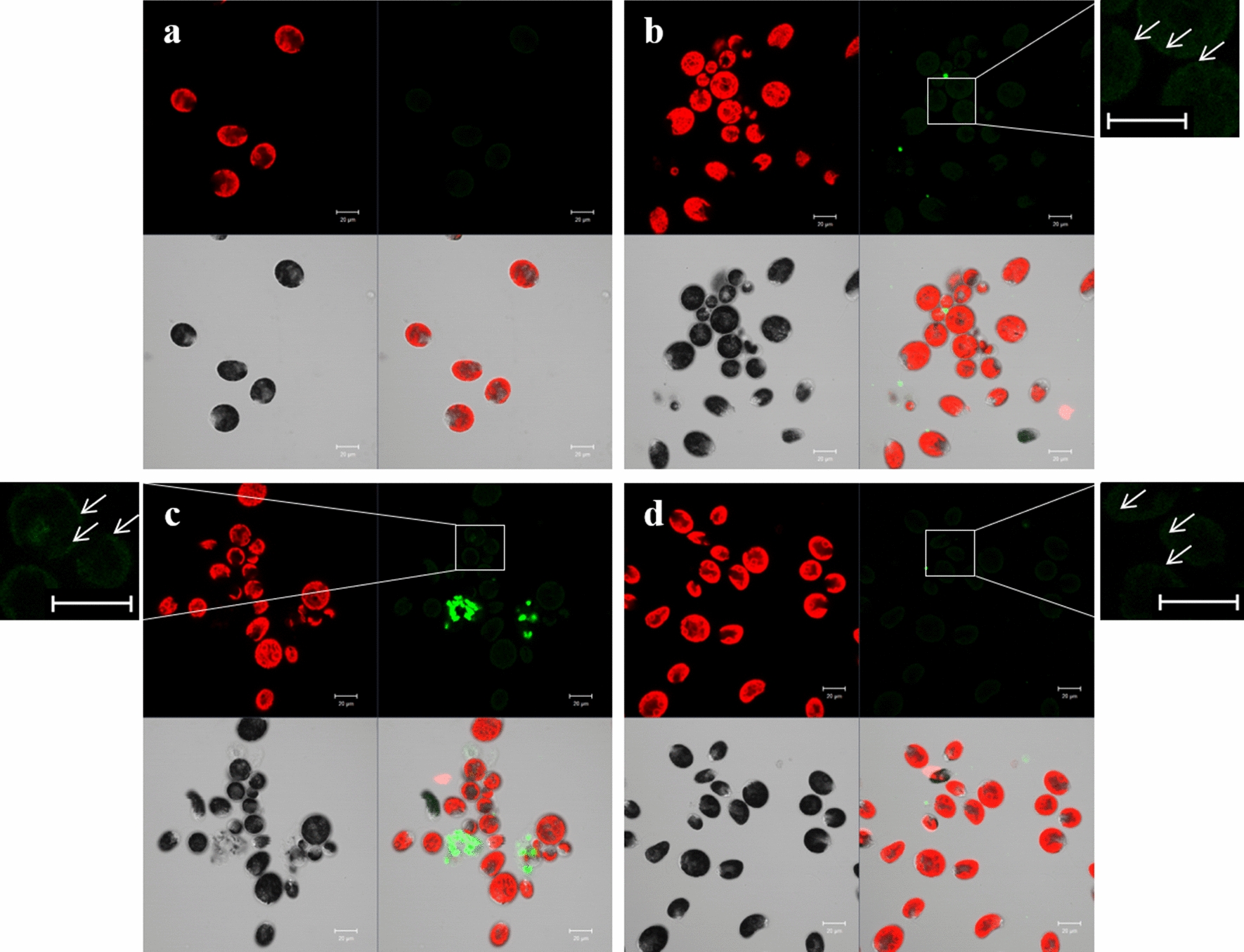
Uptake of HpEVs by *H. pluvialis* cells. The uptake of the fluorescently labeled HpEVs (green) was evident in *H. pluvialis* cells after 24 h of incubation. No stain was revealed in the untreated cells (Scale bar = 10 µm). **a** *H. pluvialis* cells treated with PBS. **b** *H. pluvialis* cells treated with HpEVs-1. **c** *H. pluvialis* cells treated with HpEVs-2. **d** *H. pluvialis* cells treated with HpEVs-3. White arrows indicated the stained HpEVs

First, cellular uptake of HpEVs by *H. pluvialis* cells was investigated using a confocal microscope. The fluorescence images revealed that single HpEVs (small green spots) were close to the cytomembrane or the cell wall, while the agglomerate HpEVs (big green spots) were on the outside of *H. pluvialis* cells, indicating that the single HpEVs could be absorbed by the cells, while the agglomerate HpEVs could not. Single HpEVs from all three growth stages could be absorbed into *H. pluvialis* cells (Fig. 2).

#### HpEVs affect the growth and metabolite synthesis *of H. pluvialis*

**Fig. 3:**
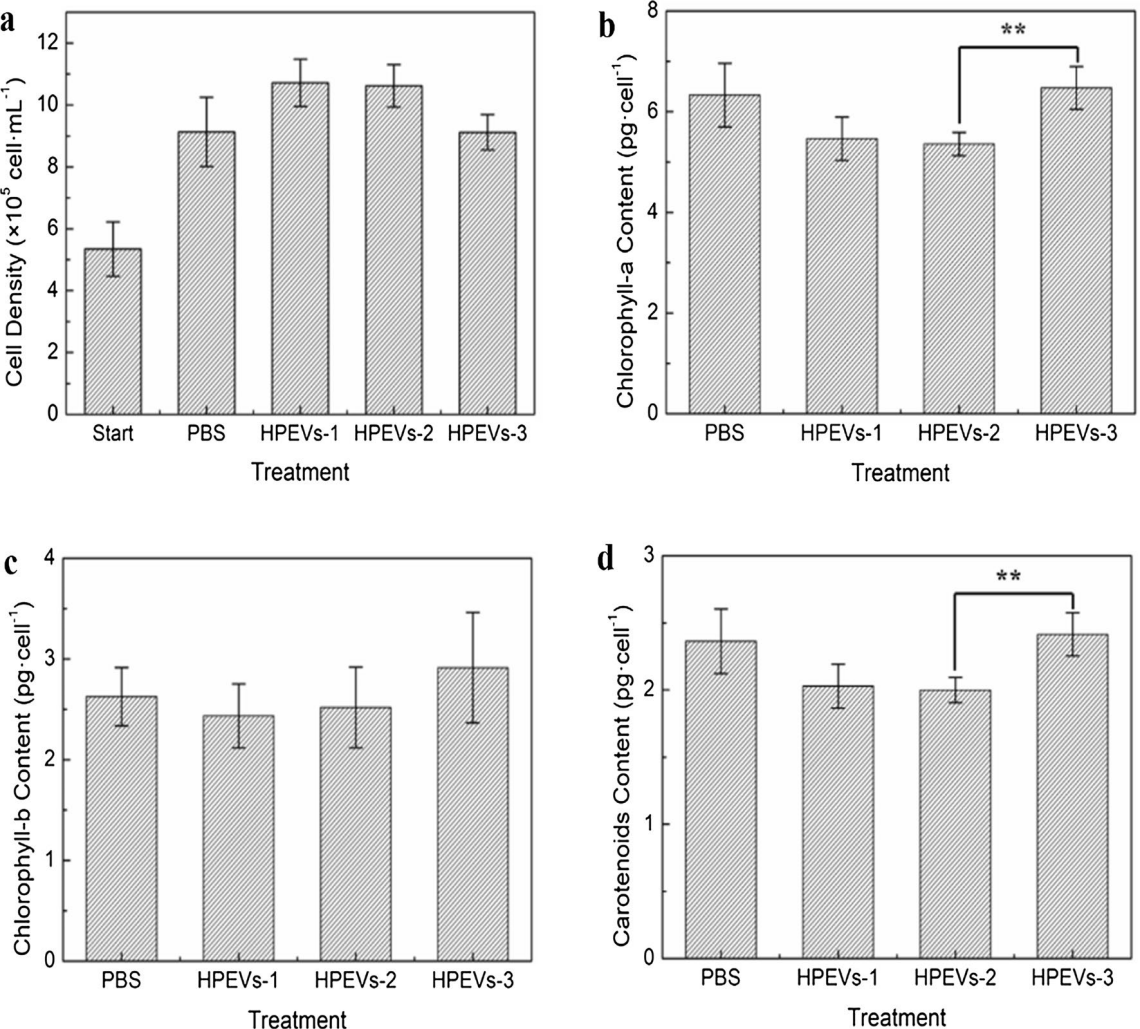
Effects of HpEVs on cell growth and single cell pigment content of *H. pluvialis*. **a** Effects of HpEVs on growth of *H. pluvialis*. **b** Effects of HpEVs on single cell chlorophyll-a content of *H. pluvialis*. **c** Effects of HpEVs on single cell chlorophyll-b content of *H. pluvialis*. **d** Effects of HpEVs on single cell carotenoids content of *H. pluvialis*. For all figures, error bars indicate standard deviation of triplicate measurements (*p < 0.05. **p < 0.01; one-way analysis of variance)

The influence of HpEVs on *H. pluvialis* growth was further studied, and the results revealed that there were no significant differences between three HpEVs treated groups though HpEVs-1 and HpEVs-2 improved the growth of vegetative *H. pluvialis* cells to 10.72–10.62 × 10^5^ cells mL^−1^ after 2 days of culture (*p* < 0.05; Fig. 3a). In addition, high level of single-cell pigments was observed in HpEVs-3 treatment after 2 days of incubation (Fig. 3b, c, d). Low levels of single-cell pigments were observed in HpEVs-1 and HpEVs-2 treatments, showing approximately 20% lower than that in HpEVs-3 treatment (Fig. 3b, c, d). Interestingly, the carotenoids’ content in the cells treated with HpEVs-3 was significantly higher than that treated with HpEVs-1 and HpEVs-2 (Fig. 3d). The single-cell astaxanthin content in the cells treated with HpEVs-3 was up to 2.66 ± 0.16 pg cell^–1^, which was approximately 19.8% higher than that in the cells treated with HpEVs-1. The highest content of FAs was observed in the cells treated with HpEVs-3 with 192.41 ± 10.47 mg *g*^–1^, whereas the treatment with HpEVs-1 resulted in the lowest content of FAs (Table 2). These results might imply that both HpEVs-1 and HpEVs-2 are more conductive to the growth, while HpEVs-3 is more conductive to the accumulation of metabolic products in *H. pluvialis*.

**Table 2:** Effects of HpEVs on single cell astaxanthin content and total fatty acid content of *H. pluvialis*

Component\treatment	Control (PBS)	HpEVs-1	HpEVs-2	HpEVs-3
AST content (pg cell^−1^)	2.73 ± 0.22^a^	2.22 ± 0.28^b^	2.33 ± 0.16^ab^	2.66 ± 0.16^ab^
Total FA Content (µg mg^−1^)	188.74 ± 11.97	173.24 ± 19.77	183.76 ± 12.85	192.41 ± 10.47

#### Inhibition of HpEVs release affect the growth and metabolite synthesis of *H. pluvialis*

**Fig. 4:**
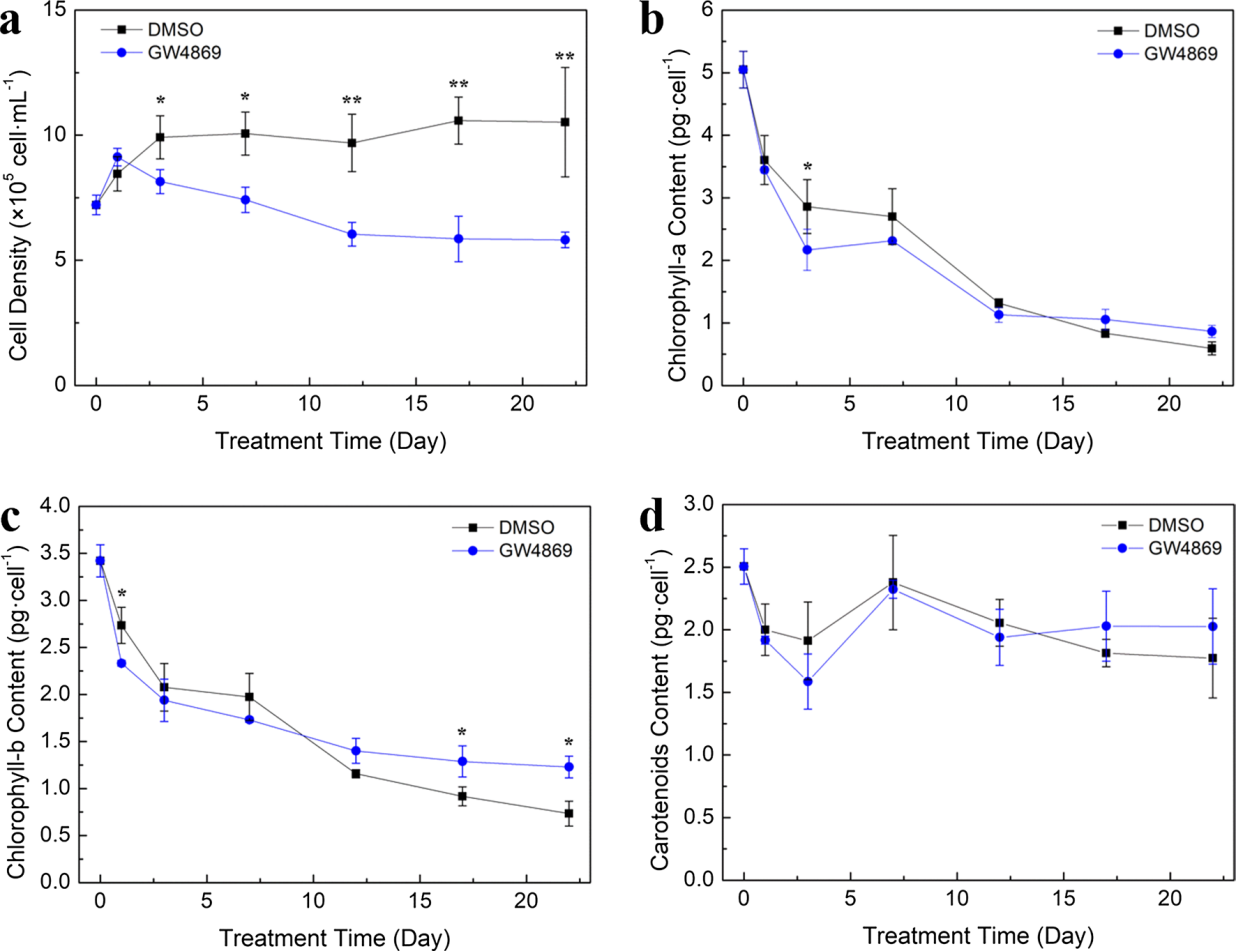
Effects of GW4869 on growth and single cell pigment content of *H. pluvialis*. **a** Effects of GW4869 on growth of *H. pluvialis*. **b** Effects of the GW4869 on single cell chlorophyll-a content of *H. pluvialis*. **c** Effects of the GW4869 on single cell chlorophyll-b content of *H. pluvialis*. **d** Effects of the GW4869 on single cell carotenoids content of *H. pluvialis*

The influence of GW4869 on the growth, physiology, and metabolism of *H. pluvialis* was studied because GW4869 could inhibit the secretion of exosomes in cells. Significant difference in *H. pluvialis* cell growth pattern was observed between the control and GW4869 treatment. GW4869 significantly improved the growth of *H. pluvialis* during the 1st day of the cultivation, with cell density being significantly higher than that of the control. Subsequently, a linear inhibition of cell growth from day 1 to day 12, followed by a stationary cell growth, was observed in the GW4869 treatment. The lowest growth rate of 5.81 ± 0.31 × 10^5^ cells mL^–1^ was achieved, which was approximately 81.70% lower than that observed in the control and also lower than that on day 0 (Fig. 4a).

The chlorophyll and carotenoid contents of *H. pluvialis* cells were analyzed. The results revealed that single-cell chlorophyll contents, including that of chlorophyll-a and chlorophyll-b, were decreased with cultivation time. Finally, a chlorophyll-a content of 0.87 ± 0.10 pg cell^–1^ was achieved, which was approximately 45.76% higher than that of the control (Fig. 4b). The final chlorophyll-b content in the GW4869 treatment was 1.23 ± 0.11 pg cell^–1^, which was approximately 68.49% higher than that of the control (Fig. 4c). Compared with the control, cells treated with GW4869 had a higher carotenoid content during the cultivation period from day 17 to day 22. Single-cell carotenoid content in the GW4869 treatment was 2.03 ± 0.30 pg·cell^–1^ after 22 days, which was approximately 14.69% higher than that in the control (Fig. 4d). Astaxanthin and total FA contents in *H. pluvialis* cells treated with GW4869 were measured after the incubation. The results revealed that the astaxanthin content of *H. pluvialis* treated with GW4869 was 7.09 ± 0.50 mg g^–1^, which was significantly lower than the control (Table 3). A lower FA content of 225.96 ± 15.36 mg g^–1^ was observed in the GW4869 treatment, which was approximately 32.84% lower than that in the control. Therefore, the inhibition of EV release may suppress the growth of *H. pluvialis* and reduce the accumulation of astaxanthin and total FAs at the same time.

**Table 3:** Effects of GW4869 on astaxanthin and total fatty acid content of *H. pluvialis*

Component\treatment	Control (DMSO)	GW4869
AST content (mg g^−1^)	8.27 ± 0.39^a^	7.09 ± 0.50^b^
Total FA content (mg g^−1^)	300.17 ± 17.33^a^	225.96 ± 15.36^b^

### Profiling and annotation of miRNAs in HpEVs

#### Identification and characterization of miRNAs in HpEVs

To investigate the expression profile of miRNAs from HpEVs, a total small RNA was extracted and analyzed from the HpEVs of the three growth stages of *H. pluvialis*. The results revealed that the HpEVs contained a considerable number of RNA sequences shorter than 100 nt in length (Additional file 1: Figure S1), confirming the enrichment of miRNA-loaded HpEVs in the cultivation media of *H. pluvialis*. A total of 74.28 million (M) raw reads were generated in the six small RNA libraries from three growth stages of HpEVs, and each small RNA library generated over 11.59 M raw reads (Additional file 1: Table S1). Clean reads of the six samples were more than 11,393,000, and the useful reads were all over 5,599,000 (Additional file 1: Table S2). Finally, a total of 163 mature miRNAs, including 93 known miRNAs and 70 novel miRNAs, were identified in the six libraries (Additional file 1: Table S3, Additional file 2: Table S4).

**Fig. 5:**
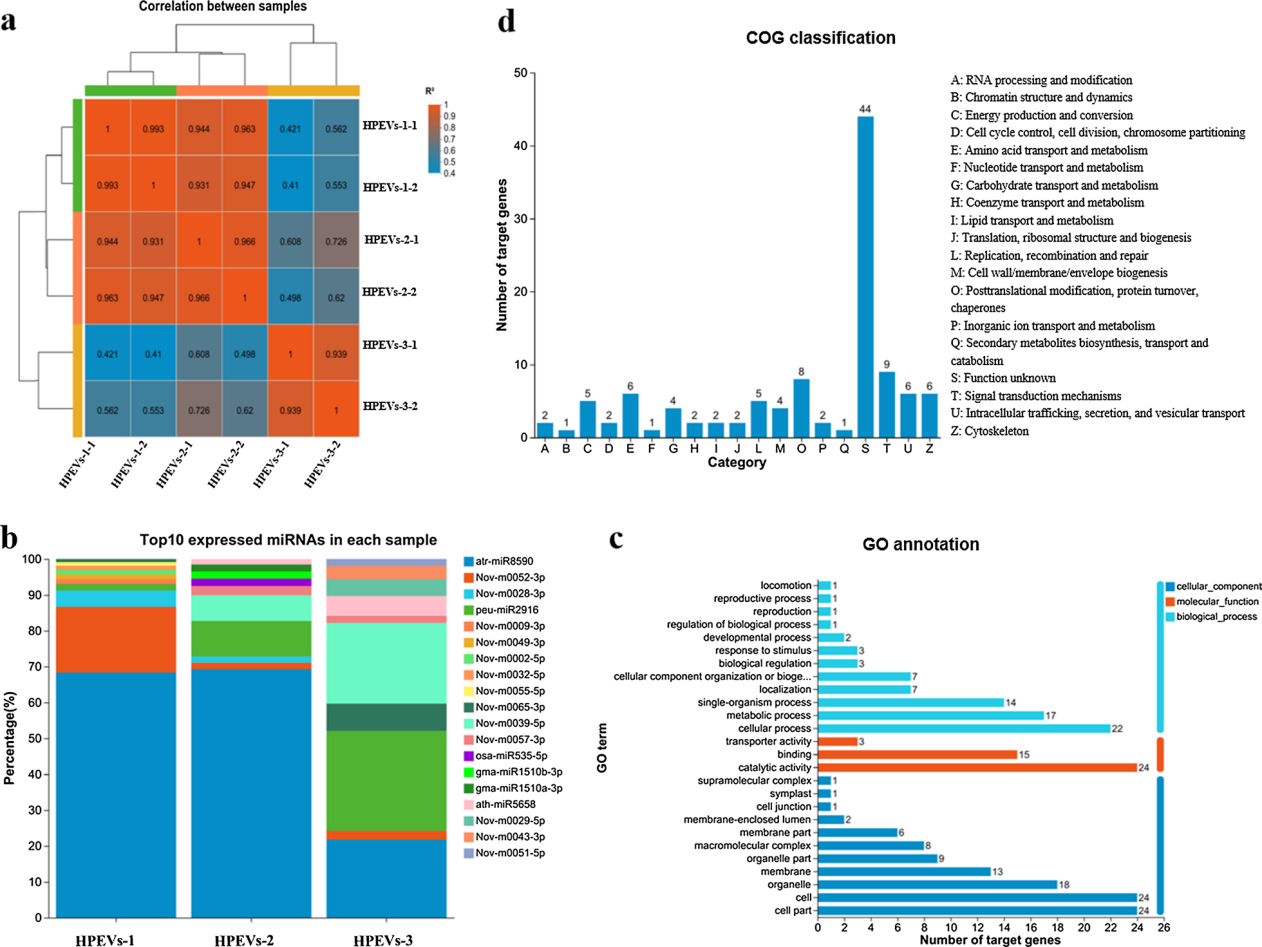
Expression pattern of the total miRNAs in three stages of HpEVs and bioinformatic analysis of the target genes of TOP 10 most highly expressed HpEV miRNAs. **a** Principal component analysis (PCA) of miRNAs from three stages of HpEVs. **b** The TOP 10 most highly expressed miRNAs to three stages of HpEVs. **c** GO annotation of the TOP 10 most highly expressed HpEV miRNAs’ target genes. **d** COG functional classification of the TOP 10 most highly expressed HpEV miRNAs’ target genes

Principal component analysis (PCA) indicated that miRNA expression profile of the HpEVs exhibited a clear stage-specific pattern (Fig. 5). Two major patterns were identified, one representing the HpEVs-1 and HpEVs-2 stages and the other representing the HpEVs-3 stage (Fig. 5a), which corresponded to the growth stages of the *H. pluvialis* cells. According to the results, a few species of miRNAs represented the majority of the whole expressed HpEVs miRNAs, and the top 10 unique miRNAs with the highest expression levels accounted for more than 68.7% of the total read counts of all identified miRNAs (Additional file 2: Table S4). The unified set of the top 10 unique miRNAs corresponded to 9 families and 35 unique miRNAs, and most miRNAs belonged to the Nov-family (Fig. 5b, Additional file 2: Table S4). Among these miRNAs, peu-miR2916, ath-mi5658, bdi-miR5054, gma-miR1510a, gma-miR1510b, gma-miR6300, osa-miR5072, Nov-m0039, and Nov-m0065 exhibited a similar expression pattern, that is, upregulated during growth stage changing from green vegetative motile stage to the red nonmotile cyst stage. In the meantime, a downregulated expression of atr-miR8590, bdi-miR5058, Nov-m0028, and Nov-m0052 was detected during the stage change phase. Therefore, the abundance of different miRNAs in HpEVs might indicate the potential regulatory function of HpEVs in the recipient cells, and the cells should uptake the HpEVs in advance.

#### Prediction the target genes of HpEV miRNAs

A total of 1473 target genes were predicted for the miRNAs, and 141 target genes were predicted for the top10 expressed miRNAs. GO annotation classified the top 10 target genes into several crucial BP terms, such as “cellular process,” “metabolic process,” “single-organism process,” “signal transduction,” and the adaptation of microalga cells to stress (Fig. 5c). The important MF terms of the highly enriched target genes were classified as “catalytic activity,” “binding,” and “transporter activity” (Fig. 5c). COG annotation of these target genes revealed that the important COG categories were “signal transduction mechanisms,” “post-translational modification, protein turnover, chaperones,” “intracellular trafficking, secretion, and vesicular transport,” “amino acid transport and metabolism,” and “cytoskeleton” (Fig. 5d). The enrichment of these physiology, metabolism, and signal transduction-related miRNAs in the HpEVs may imply their role in intercellular communication among the *H. pluvialis* cells.

#### Differentially expressed HpEV miRNAs

**Fig. 6:**
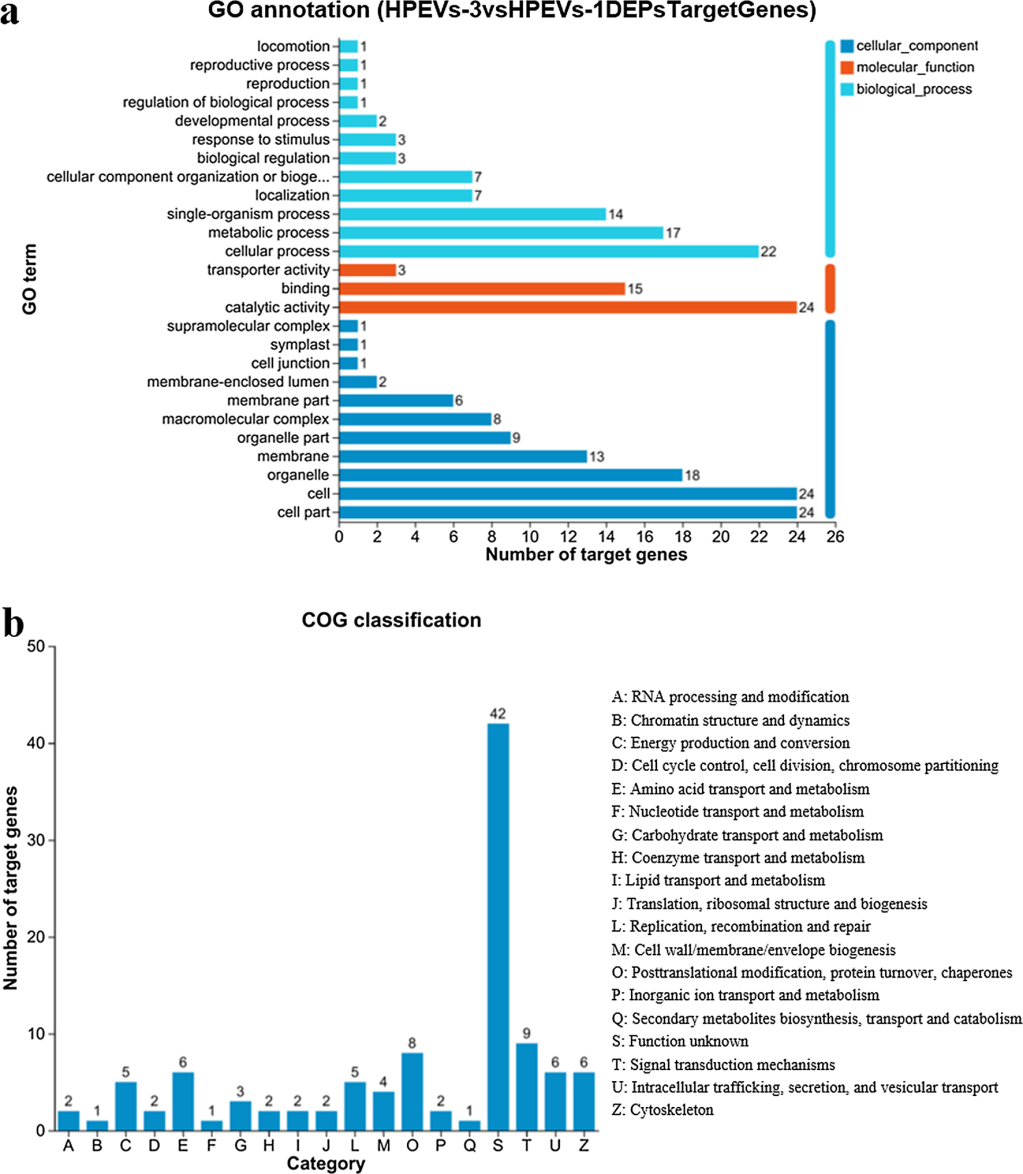
Bioinformatic analysis of the target genes of differentially expressed miRNAs between HpEVs-3 and HpEVs-1. **a** GO annotation of the differentially expressed miRNAs’ target genes. **b** COG functional classification of the differentially expressed miRNAs’ target genes

In this study, target prediction and functional annotation analyses of the differentially expressed HpEV miRNAs between HpEVs-1 and HpEVs-3 were conducted, and a total of 12 differentially expressed miRNAs were identified. Of these, 3 were upregulated, and 9 were downregulated (Additional file 1: Table S5). Target prediction of the differentially expressed miRNAs led to the identification of 144 target genes of the downregulated miRNAs, while no target genes of the upregulated miRNAs were identified (Fig. 6). The results of GO annotation of these target genes were consistent with those of the top 10 most highly expressed HpEV miRNAs (Fig. 6a). COG classification of the downregulated miRNA target genes of the HpEVs-3 versus HpEVs-1 group revealed the highly classified clusters to be “signal transduction mechanisms,” “post-translational modification, protein turnover, chaperones,” “amino acid transport and metabolism,” “intracellular trafficking, secretion, and vesicular transport,” and “cytoskeleton” (Fig. 6b). The other identified COG clusters were “cell wall/membrane/envelope biogenesis,” “cell cycle control, cell division, chromosome partitioning,” “lipid transport and metabolism,” and “secondary metabolites biosynthesis, transport, and catabolism,” which were the important HpEV functions that we focused on. The analysis of differentially expressed miRNAs of different growth stages may reveal that the microalga cells with different physiological statuses sort diverse miRNAs into the HpEVs. Subsequently, target genes of the differentially expressed miRNAs, related to physiology and metabolism of the microalga cells, would be differently regulated in the recipient *H. pluvialis* cells, resulting in the changes in growth and physiology. Therefore, HpEVs extracted from the red nonmotile cysts exhibit positive effects on signal transduction, protein biosynthesis, secondary metabolism, and cell wall synthesis of *H. pluvialis* cells, as revealed by the miRNA profiling.

### Functional analysis of HpEVs on *H. pluvialis*

To explore the biological functions of HpEVs on *H. pluvialis* in-depth, the expression levels of 17 identified genes involving in cell cycle controlling, cell wall construction, fatty acid biosynthesis and carotenoid biosynthesis pathways in *H. pluvialis* were investigated using qRT-PCR method.

#### The effects of HpEVs on gene expression in *H. pluvialis*

The results shown that, most of the genes were downregulated in the cells treated with HpEVs-1, while most of them were upregulated in the cells treated with HpEVs-3 (Table 4). The expression level of cell division cycle protein 45 gene (*CDC45*), which plays a role in the initiation of DNA replication, revealed no significant difference among the cells treated with PBS and three HpEVs though the lowest level was observed in HpEVs-1 treated group. The expression levels of cell cycle control genes, including type A cyclin gene (*CYCA*) and type B cyclin gene (*CYCB*), significantly increased from HpEVs-1 to HpEVs-3 treatments for 5- to 6-fold. In terms of the cell wall biosynthesis-related genes, including cellulose synthase gene (*CS*), O-Glycosyl hydrolases gene (*OGH*), glucose/GDP mannose dehydrogenase gene (*G/GMND*), mannitol dehydrogenase gene (*MTD*), and glucose-6-phosphate 1-epimerase gene (*GPE*), similar expression patterns with that of the cell cycle control genes were revealed. In particular, the *CS*, *OGH*, and *MND* genes were significantly upregulated with approximately 3- to 9-fold treated with HpEVs-3 in comparison with HpEVs-1. The above results of cell wall biosynthesis-related genes indicated HpEVs of the late growth stages might play roles in encyst formation during *H. pluvialis* life cycle. Expression levels of the four genes involved in the FA biosynthesis pathway, including biotin carboxylase gene (*BC*), acyl carrier protein gene (*ACP*), stearoyl ACP-desaturase gene (*SAD*), and ω-3 fatty acid desaturase gene (*FAD*), were approximately 2- to 7-fold higher in the HpEVs-3 treated group than in the HpEVs-1 treated group, suggesting that HpEVs-3 positively affect FA biosynthesis. Moreover, expression levels of the key enzyme genes involved in the carotenogenic biosynthesis pathway, including phytoene synthase gene (*PSY*), lycopene β-cyclase gene (*LCY*), carotenoid hydroxylase gene (*BKT*), and β-carotene hydroxylase gene (*CRTR-B*), were significantly increased from HpEVs-1 to HpEVs-3 treatments. Expression levels of *PSY*, *LCY*, and *CRTR-B* were approximately 3- to 9-fold higher in the cells treated with HpEVs-3 than in those treated with HpEVs-1. Therefore, it is indicated that HpEVs-3 improves the carotenoid content by upregulated the expression of carotenogenic-related genes.

**Table 4:**
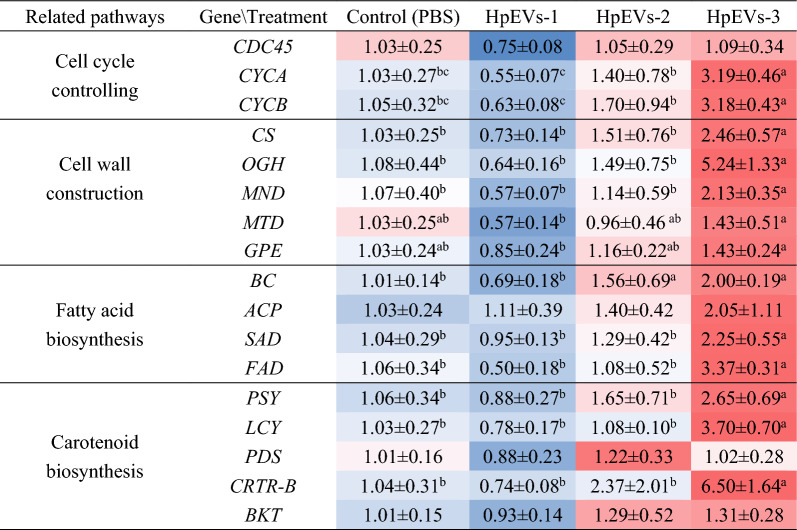
Effects of HpEVs on expression level of key genes in specific metabolic pathways

#### The effects of HpEVs secretion inhibition on gene expression in *H. pluvialis*

The effects of GW4869 on the expression levels of 17 key genes of *H. pluvialis* are listed in Table 5. Significant difference was observed between the GW4869 and control treatments. Regarding the cell cycle control genes, expression levels of *CDC45* and *CYCB* significantly decreased in treatment initiation, and both of them were at low expression levels and had no significant differences during the incubation period. The expression of *CYCA* was also at low level though it significantly increased at day 1 and 7. As for the cell wall construction genes, the expression of *CS* and *MND* were downregulated since treatment initiation, with a low level during the incubation period. The expression of *OGH* and *MTD* rapidly increased after day 1 of the cultivation, and then kept at high levels, up to 5- to 10-fold at day 22 than that at day 0. Overall, a lower expression level was observed in the GW4869 treatment than in the control during a similar treatment period. In the GW4869 treatment, the expression of *MTD* increased from day 0 to day 7; after reaching a peak, a high level of expression was maintained. No significant difference in *GPE* expression between the treatments was observed throughout the incubation period.

**Table 5:**
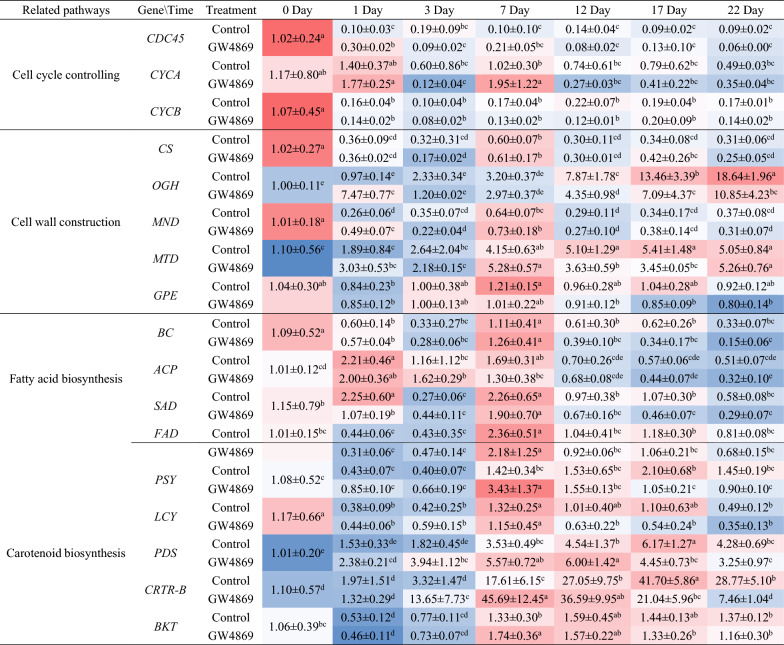
Effects of GW4869 on expression level of the key genes in specific metabolic pathways during the treatment period

Genes related to fatty acid biosynthesis, including *BC*, *ACP*, *SAD*, and *FAD,* were studied. *BC* expression decreased from day 0 to day 3, reached the highest level on day 7, and decreased from day 12 to day 22. In the GW4869 treatment, a low *BC* expression was observed during the last 10 days compared with the control treatment. The expressions of *ACP* and *SAD* were significantly upregulated after 1 and 7 days, respectively, of treatment to achieve the highest level, followed by a decrease in expression with treatment. Moreover, *ACP* and *SAD* were downregulated in the GW4869 treatment compared with the control treatment during the last 15 days, that is, from day 7 to day 22. The expression level of *FAD* significantly decreased in the first 3 days, followed by an increase to achieve the highest level on day 7 and a reduction to a consistent level on day 0. Overall, the FA biosynthesis-related genes exhibited downregulation in cells treated with GW4869.

Treated with GW4869, the expression levels of carotenogenic genes were first shown a slight decrease on day 1, increased on day 7 and 12, and finally declined on day 22 when compared with control group. The expression level of *PSY* in treated group reached the highest on day 7 with 1.4-fold to the control group, while decreased by 0.61 times on day 22. A low level of *LCY* expression was observed during the whole treatment period, and no significant differences were observed between the treatments. The expression level of *BKT* exhibited no significant differences between the treatments during the same period except for the 7 days of treatment, in which the expression level was significantly higher in the GW4869 treatment than in the control treatment. The expression levels of *PDS* and *CRTR-B* exhibited a similar change pattern. In the GW4869 treatment, *PDS* and *CRTR-B* were gradually upregulated during the first 7 days, followed by a downregulation. The expression levels of these genes were higher in the GW4869 treatment than in the control treatment during the first 12 days, while with a decrease from day 17 to day 22. In general, inhibition of HpEVs secretion showed negative effect on the growth, cell wall construction, fatty acid biosynthesis, and carotenoid biosynthesis in the long term.

## Discussion

The determination of origin and functions of plant EVs is essential to understanding plant cell-to-cell communication, which increases the fundamental knowledge of plant physiology and of processes that may be used in their beneficial manipulation [18, 22, 30, 36]. EVs have gained widespread attention, because they are a rich source of molecules with therapeutic and biomarker potential, among which miRNAs have been considered as key and most well-studied molecules in EVs [5–7, 23, 30, 33, 36].

In this study, characterization and function verification of the EVs extracted from the cultivation media of *H. pluvialis* (HpEVs) at three growth stages were performed. HpEVs were successfully isolated from the cultivation media of the microalga at three growth stages. It was observed that the extracted HpEVs were typical spherical or quasi-spherical bilayer-enclosed nanovesicles with sizes ranging from 50 to 200 nm, a result consistent with that in edible plants and microalgae in the previous studies [17, 24, 38–42]. The amount of the isolated HpEVs increased with the cultivation period, which might because stability of these bilayer-enclosed nanovesicles enabling them to be continuously accumulated in the culture media.

Numerous reports have investigated EV uptake by multiple types of target cells, and would play a fundamental role as extracellular messengers to mediate cell-to-cell communication, even cross-species and cross-kingdom [26, 29, 30, 45, 46]. In this study, the cellular uptake of HpEVs by *H. pluvialis* cells was confirmed using fluorescent staining and visualization. The influence of HpEVs on *H. pluvialis* was further determined, and the results demonstrated that HpEVs extracted from the cultivation media of the green cell stages were more functional in promoting growth, whereas red cyst-derived HpEVs were more functional in regulating metabolite synthesis. With inhibition of the secretion of EVs by *H. pluvialis* cells using GW4869, improvement in cellular growth on the 1st day of cultivation was observed. The inhibition was more profound with the prolonged treatment time, even leading to *H. pluvialis* cell death. Meanwhile, accumulation of carotenoids and higher single-cell chlorophyll contents were achieved under the HpEV secretion inhibition condition. However, astaxanthin and fatty acid accumulation of the *H. pluvialis* cells were inhibited at the same time. These results indicated that growth and primary or secondary metabolism were negatively influenced under the HpEV secretion inhibition condition. In the meantime, a higher cellular photosynthesis rate was observed.

miRNAs within EVs are suggested to regulated expression of the target genes in recipient cells at the post-transcriptional level [7, 8, 12]. In this study, a large number of miRNAs were detected in the isolated HpEVs, and similarities and differences in miRNA species and expression levels were determined in different HpEVs extracted from the microalgal cells at three different growth stages. Overall, a total of 163 mature miRNAs, including 93 known miRNAs and 70 novel miRNAs, were identified. Based on the bioinformatics analysis, these miRNAs were considered to be involved in diverse aspects of microalgal growth and physiology. For example, peu-miR2916 can be detected in many plants and plant-derived exosome-like nanoparticles and are abundant plant miRNAs predicted to target human genes [17, 47]. The target genes of miR5658 were involved in plant development, signal transduction, metabolic pathways, disease resistance, and environmental stress response [48]. Downregulation of miR5054 was observed under drought stress, which was speculated to promote *ZmOSCA2.4* expression and stress resistance [49]. Cui et al. [50] suggested that gma-miR1510 plays a potential regulatory role in soybean defense against *Phytophthora sojae* infection, and the expression levels of gma-miR1510 were downregulated gradually by *P. sojae* treatment. In this study, atr-miR8590 was a highly abundant miRNA in all three HpEVs. atr-miR8590 was first described in *H. pluvialis* and exhibits a physiological function in promoting the growth of microalga cells. Target genes of the miRNAs in HpEVs were associated with growth, metabolism, and stress response. The results of DEP analysis indicated two obvious stage-specific patterns of HpEV miRNAs. A total of 12 miRNAs were expressed differently with statistical significance, including 9 downregulated miRNAs and 3 upregulated miRNAs. The miRNA profiling was performed using COG annotation, which has been previously reported in other EVs [4, 51]. Results indicated that the highly classified COG clusters of the target genes of the downregulated miRNAs were related to signal transduction, protein biosynthesis, secondary metabolism, cell division, and cell wall synthesis, thereby demonstrating the positive regulatory functions of the red cyst-derived HpEVs on these related genes.

There was no significant difference between the HpEV treatments in the expression level of *CDC45*, which is related to DNA replication of the recipient *H. pluvialis* cells [52]. Cyclins are critical regulators of cell cycle progression; *CYCA* is responsible for the G1/S phase transition, and *CYCB* regulates the G2/M transition [53–57]. The expression patterns of *CYCA/B* in response to HpEV treatments indicated that HpEVs extracted from late growth stages of microalga significantly promoted phase transition of the recipient *H. pluvialis* cells in comparison with that of HpEVs extracted from the early growth stages. Interestingly, expression level of these cell cycle regulation genes exhibited an opposite change pattern to that of *H. pluvialis* cellular growth, indicating that the highly expressed cyclin genes under the influence of HpEVs extracted from the late growth stages encourage cell division, whereas DNA replication was not promoted by the corresponding HpEVs. Thus, cellular growth was inhibited by HpEVs extracted from the late growth stages. The cell wall biosynthesis-related genes were upregulated when exposed to salicylic acid and high light stresses due to mannose formation in the aplanospore cell wall of *H. pluvialis* [58]. In this study, the cell wall biosynthesis-related genes were upregulated when treated with HpEVs extracted from the late growth stages compared with HpEVs extracted from the early growth stages, demonstrating that that HpEVs extracted from the late growth stages promoted aplanospore cell wall formation in the recipient *H. pluvialis* cells, whereas HpEVs extracted from the green vegetative cells inhibited aplanospore cell wall formation. Both fatty acids and astaxanthin biosynthesis in *H. pluvialis* were also promoted by HpEVs extracted from the late growth stages and inhibited by HpEVs extracted from the early growth stages [59–61]. These results revealed that HpEVs of different stages exhibited variant influence on the *H. pluvialis* recipient cells after being uptaken, and the influence was linked to the physiological state of the donor *H. pluvialis* cells, enabling cell–cell communication. In conjunction with early hypothesis favored bioactive roles of exosomes in maintenance of normal physiology. HpEVs were hypothesized to modulate cell physiological state in normal conditions, and to expel harmful cellular constituents from cells under stress conditions [62–64]. The harmful cellular constituents in HpEVs then play a role in the regulation secondary metabolism of the ingested cells.

It was also indicated that DNA replication and cell division of the recipient cells were positively influenced when HpEV secretion was inhibited during the early treatment period, whereas a negative influence was observed during the late treatment period. The inhibition of cell wall formation in recipient cells was observed when HpEV secretion was inhibited during the entire incubation period. Fatty acid and astaxanthin biosynthesis increased when HpEV secretion was inhibited during the early treatment period, while an inhibitory effect was observed during the late treatment period. The expression levels of specific pathway genes in *H. pluvialis* were consistent with the physiological properties of the treated cells, indicating that short-term inhibition of HpEV secretion provokes cell growth and secondary metabolism in accordance with HpEV function. However, long-term inhibition of EV secretion inhibited cellular growth, cell wall formation, and primary and/or secondary metabolism. It was indicated that microalgal-derived EVs served functions in providing microorganisms in the microenvironment with a source of energy and nutrient [65, 66]. Therefore, when the release of HpEVs was inhibited by GW4869, the transferring of energy and nutrient out of the cells through EVs secretion was disrupted. It was hypothesized that the growth and secondary metabolism of *H. pluvialis* cells were promoted by these entrapped cargos of HpEVs, which accumulated in the microalgal cells for they could not be released with HpEVs. While continuous inhibiting HpEVs’ secretion suppressed the growth of cells along with the harmful cellular constituent accumulated in the cytoplasm [64]. These results were consistent with early hypotheses that EVs involve in many physiological functions and processes and carry out diverse range of functions depending on their variant cell states [63].

**Fig. 7:**
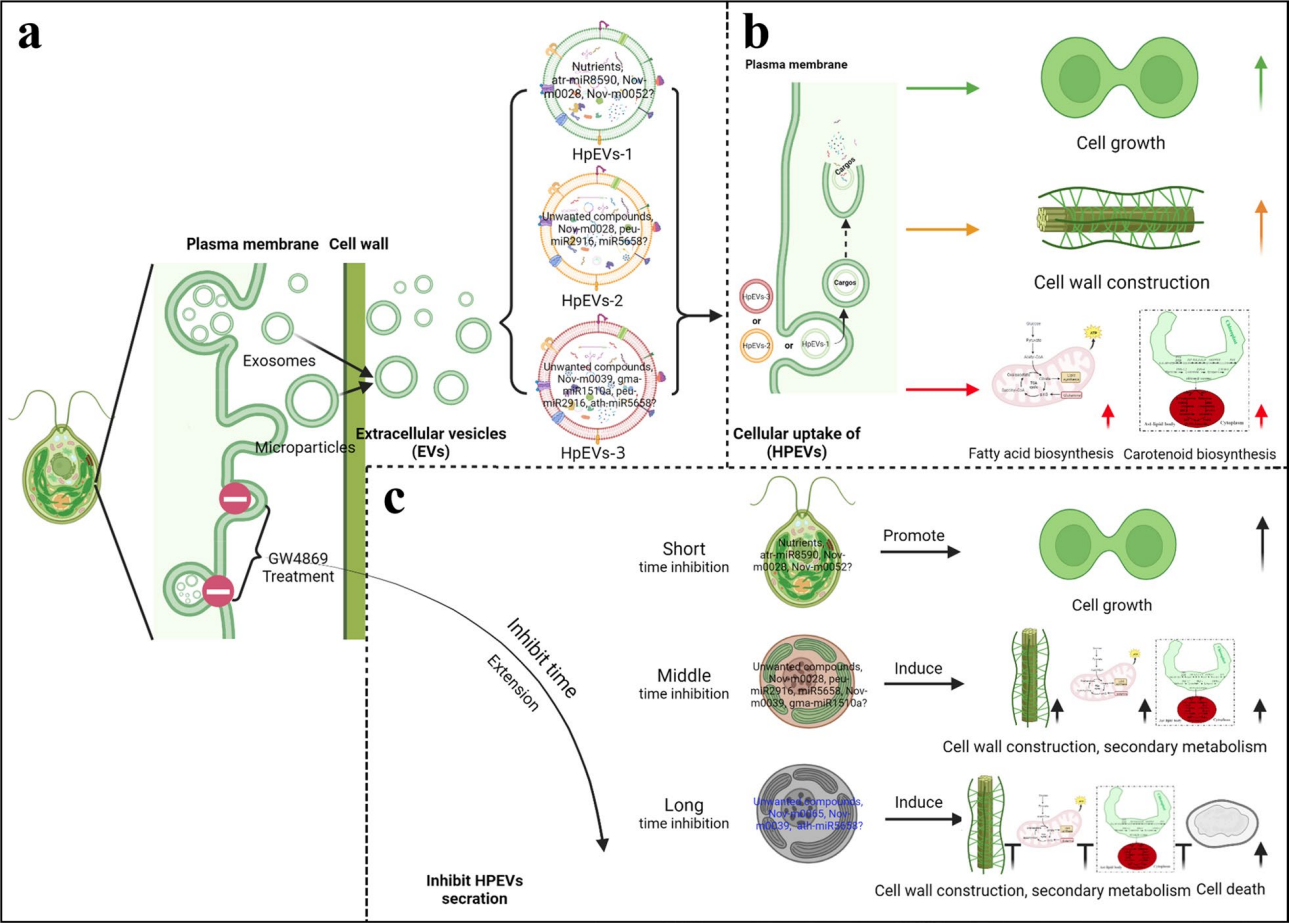
Extracellular vesicles (EVs) in *H. pluvialis*. **a** Origin and secretion of EVs in *H. pluvialis* (HpEVs). **b** HpEVs regulate the growth and metabolite synthesis of *H. pluvialis*. **c** Inhibiting HpEVs’ release affect the growth and metabolism of *H. pluvialis*

To summarize (Fig. 7, generated with BioRender, https://www.biorender.com/), *H. pluvialis* cells sort distinct miRNAs into the HpEVs, which reflects the physiological state of the secretion microalgal cells, thereby endowing these vesicles with cell type-specific biological functions, as previously described [23, 25, 67]. These HpEVs access the extracellular space through unconventional secretion and could be isolated and purified from the complex culture media [42, 68]. miRNA profiling demonstrates heterogeneous of the HpEV-miRNAs, whereas the bioinformatics analyses suggest their involvement in cell proliferation, cell metabolism, and cell response to stress [4, 9, 10, 23]. HpEVs derived from different growth stages of donor *H. pluvialis* cells might exert variant effects on recipient cells after transferring their cargoes into these target cells. The practical impacts of HpEVs are either related to their overall complex cargo signature or to certain individual biologically active macromolecules [4]. Our findings indicate that HpEVs can be uptake by *H. pluvialis* cells, and they have regulatory and functional effects on cell growth and physiological features of the recipient cells, which is consistent with functions of the target genes of their miRNA cargoes. Thus, it is highlighted that the secretion and ingestion of HpEVs is a way of cell-to-cell communication among *H. pluvialis* cells [25]. The analysis of HpEV secretion inhibition delineates consistent results with early hypothesis favoring the notion that exosomes maintain cellular homeostasis by excreting harmful or unusable cellular constituents from cells through exosome secretion [15, 62, 64, 69]. However, the short-term inhibition of HpEV secretion results in the accumulation of functional cellular components of HpEVs, thereby altering the biological response of these cells at the molecular level. These results are in accordance with previous reports that EVs play a role in regulating intercellular communication [69].

The ability to produce multiple bioproducts within a single production facility make the commercial realization of *H. pluvialis* as a potential feedstock for multiple-product biorefining, which allows the maximum utilization of the feedstock, minimizing waste generation and improving the sustainability of the process while promoting the concept of a circular bioeconomy [43, 44]. The investigation of high-quality HpEVs manufacturing from cultures of this microalgae offers us an additional approach for *H. pluvialis* utilization more efficiently. Despite the high biotechnological potential of *H. pluvialis*, a mature two-stage culture strategy was designed for large-scale *H. pluvialis* cultivation and astaxanthin production, and satisfactory attempts for one-stage cultivation of *H. pluvialis* were also reported by researchers [44]. And, renewable and scalable bioprocess for the scalable production of EVs from microalgae cultures with high quality also highlighted technical feasibility of HpEVs’ production suitable for diverse scientific and industrial exploitations [40–42]. It becomes evident that large-scale strategies are currently available for the commercial-scale production of HpEVs for diverse scientific and industrial exploitations. Moreover, the existing researches of *H. pluvialis* could undoubtedly provide research basis for studies of biological properties and application approaches of HpEVs as a drug-delivery nano-platform. In addition, results observed in this study offer the potential of HpEVs the bioengineering possibility for specifically application, because cargoes and functions of different stages of HpEVs are also obviously different. According to the results of biological properties and miRNA profiling of HpEVs, a research basis was provided for the engineering of HpEVs like-minded and synergistic with its own nature. In general, all the above advantages make HpEVs greater application potential in comparison with other microalgae-derived EVs.

## Conclusion

The observations and suggestions made herein interpret the functions of EVs in microalgal intercellular interaction, thereby highlighting the importance of EVs and their commercial application. In addition, the results established the potential functions of microalgae-derived EVs in trophic-level interactions and population substitution in natural environments, deepening our understanding of the stabilization mechanism of the aquatic ecosystem. Full of various kinds of bioactive molecules in edible microalgae-derived EVs highlight them the potential therapeutic approach for human health-promoting activities. Controllable characterization of HpEVs at a controlled purity level will emphasize microalgae-derived EVs as promising therapeutic factors or drug-delivery nanoparticles in medical applications. However, further studies on the optimization of upstream and downstream processing of microalgal EVs, including screening and genetically engineering suitable strains of microalgae for scaling up production of EVs, microalgal cultivation and quantification, large-scale isolation protocols for pure and concentrated EVs, in-depth mechanistic insights into how their significant components influence processes of microlagal growth and cultivation, and their bioactive effects on human pathophysiological mechanisms, are required to derive and realize their application as a participant in clinical biotherapeutics and therapeutic drug carriers and the industrial exploitation of microalgal-derived EVs.

## Materials and methods

### Algal strain and cultivation conditions

*H. pluvialis* 192.80 was obtained from the Sammlung von Algenkulturen Culture Collection of Algae at Gottingen University and cultured in Bold Basal Medium (BBM) in 1000-mL culture flasks with working volumes of 900 mL in an illumination incubator. The cells were cultured in suspension, wherein the flasks were continuously bubbled with air filtered through a 0.22-µm Millex Syringe-driven Filter Unit (Millipore, Ireland). The culture temperature in the illumination incubator was approximately 22 ℃, light intensity was 20 µmol·m^–2 ^s^–1^, and the light-to-dark ratio was 24 h to 0 h. The culture was grown to the logarithmic phase (approximately 5 × 10^5^ cells mL^–1^). Subsequently, the cultures were pooled and evenly divided into 9 aliquots of 1000 mL each in 2000-mL Erlenmeyer flasks, which were incubated under high light (350 µmol m^–2^ s^–1^) and high sodium acetate (45 mM) conditions. The cultures were grown for 0 h, 9 h, and 48 h to achieve the different stages of *H. pluvialis* life cycle, that is, green vegetative motile stage, green nonmotile stage, and red nonmotile cyst stage. Three microalgal cultures were randomly selected for extraction of *H. pluvialis* EVs (HpEVs). Each treatment was performed in triplicates.

### HpEVs isolation from cultivation media

The culture solution of *H. pluvialis* was collected by sequentially centrifuging at 2000 × *g* for 10 min and 10,000 × *g* for 30 min to remove the microalga cells and debris. The collected supernatant was microfiltered through 0.22-µm Durapore PVDF membranes (Millipore, Ireland) using a Nalgene Vacuum suction filtration device, which was sterilized by autoclaving before use. The filtered liquor was ultrafiltered using 100 kD Amicon Ultra-15 Centrifugal Filters (Millipore, Ireland) to remove proteins with a molecular weight of < 100 kDa, followed by centrifugation at 6000 × g. Finally, the ultrafiltrate, enriched with HpEVs, was collected for each HpEV sample. The HpEVs can stably bind to membranes, and the membranes were washed with cool and sterilized PBS buffer to reduce HpEV loss. The washing PBS and ultrafiltrate were collected together, with a total volume of approximately 20 mL. Subsequently, the HpEVs were pelleted at 100,000 × *g* for 80 min and washed once with PBS; the HpEV pellets were resuspended in 100 µL sterilized PBS buffer. The HpEVs were stored at 4 ℃ for less than 7 days or immediately frozen in liquid nitrogen and stored at –80 ℃ until further analysis. In this study, HpEVs isolated from three stages of the *H. pluvialis* were sampled after high light and high sodium acetate treatment for 0, 9, and 48 h, with these HpEVs being defined as HpEVs-1, HpEVs-2, and HpEVs-3, respectively. All the above HpEV isolation procedures were performed at 4 ℃.

### HpEVs identification and quantification

The particle size of the HpEVs was determined using a ZetaPlus Particle Size Analyzer (Brookhaven Instruments Co., USA). To measure HpEV morphology, negative staining with 1% phosphotungstic acid was performed, and the vesicles were viewed using a Hitachi HT7700 120-kV transmission electron microscope (TEM) (Hitachi, Japan). The observation steps were as follows: (1) approximately 6 µL HpEV suspension was applied by dripping on the carbon side of 3.05-mm copper Formvar-carbon-coated electron microscopy grids (Head Biotechnology CO., China), and two to three grid samples were prepared for each HpEV sample; (2) the grids were placed in the dark in quiescence for 20 min for adsorption of the HpEVs onto the grids; (3) an absorbent paper was used to remove any spare droplets of the HpEVs, and the grids were air-dried for 2 min; (4) 10 µL of 1% phosphotungstic acid was added on the grids, followed by incubation in the dark for 2 min for staining of the HpEVs; (5) the dyeing liquor was removed using an absorbent paper, and the grids were placed on dry absorbent paper with the carbon side facing upward; and (6) the grids were air-dried under room temperature for more than 12 h, followed by visualization and imaging at 80 kV using a TEM.

### Effects of HpEVs on *H. pluvialis*

To verify the functions of HpEVs, two tests were performed to analyze the effects of different HpEVs (HpEVs-1, HpEVs-2, and HpEVs-3) on *H. pluvialis* cells.

First, an HpEV uptake experiment was conducted. Because EVs have a lipid bilayer membrane structure, the isolated HpEVs were labeled with PKH67 lipophilic dyes using the PKH67 Fluorescent Cell Linker Kit (Sigma-Aldrich, USA) in accordance with the manufacturer’s instructions. The labeled HpEVs were washed and resuspended in 100 µL sterilized PBS for further analyses. *H. pluvialis* cells in the logarithmic phase (approximately 5 × 10^5^ cells mL^–1^) were collected by centrifugation at 1000 × g for 5 min, followed by washing twice with fresh BBM and resuspension in fresh BBM at a cell concentration of approximately 1 × 10^6 ^cells mL^–1^. The PKH67-labeled HpEVs were added to 1 mL of *H. pluvialis* which was resuspended in fresh BBM, and incubated under continuous light with 20 µmol m^–2^ s^–1^ for 24 h at 22 ℃. Subsequently, the cells were measured using a laser-scanning microscope (Zeiss, LSM 710 NLO, Germany) with a 100X EC Plan-Neofluar 40/1.30 Oil DIC M27 Oil immersion objective lens. The confocal images were acquired using laser excitation at 405, 488, or 546 nm, and the images were integrated using Zen Software (Zeiss, RRID: SCR_018163, Germany). PKH67 emits a green fluorescence under the excitation of 488-nm light.

Second, HpEVs with a final protein concentration of 10 µg L^–1^ were added to the mid-log phase *H. pluvialis* culture. The *H. pluvialis* cultures were obtained by harvesting the cells after centrifugation at 1500 × *g* for 10 min, washing three times with fresh BBM, and resuspending in fresh BBM to achieve a cell density of approximately 5 × 10^5^ cells mL^−1^. Subsequently, 166.67 µL of HpEV suspensions diluted to a concentration of 60 µg mL^–1^ was added to 100 mL of *H. pluvialis* culture for cocultivation. The control sample was treated with the solvent, sterilized PBS, at the concentration similar to that of HpEV suspension. For facilitate comparing the growth process of *H. pluvialis* during HpEVs treatment, we defined a start process of *H. pluvialis* as treating with PBS or HpEVs for 0 h. After 2 days of cultivation, the cell concentration of the treated samples was measured, and the *H. pluvialis* cells were harvested and washed three times with sterile water; the cells were immediately frozen in liquid nitrogen and stored at –80 ℃ until further analysis. Three biological replicates of the three HpEVs and controls were constructed in this study.

### Inhibition of the HpEVs secretion

To evaluate the effects of HpEVs’ secretion inhibition on *H. pluvialis*, GW4869 (Sigma-Aldrich, USA), a neutral sphingomyelinase II inhibitor which can reduce exosome secretion of the cells, with final concentration of 10 µM was added into the cultivation system of *H. pluvialis* during late-log phase. GW4869 was initially dissolved in DMSO (Thermo Fisher Scientific, USA) to obtain a stock solution of 10 mM GW4869 before its addition to the culture supernatant (note: final DMSO concentration was 0.1%). The late log phase microalga cells were harvested by centrifugation at 1500 × *g* for 10 min, followed by washing three times with fresh nitrogen and phosphorus-deficient BBM (0N0PBBM) and resuspending in fresh 0N0PBBM to achieve a cell density of approximately 1 × 10^6^ cells mL^–1^. The culture temperature in the illumination incubator was approximately 22 ℃, light intensity was 350 µmol m^–2^ s^–1^, and the light-to-dark ratio was 24 h to 0 h. The cells were treated for 0, 1, 3, 7, 12, 17, and 22 days, and the cell concentration was measured. The cells were also harvested and washed three times with sterile water, immediately frozen in liquid nitrogen, and stored at –80 ℃ until further analysis. The control sample was treated with the solvent, DMSO, at a concentration of 0.1%, to achieve a concentration similar to that of GW4869 liquor. Three biological replicates of the control and GW4869 treatment samples were constructed in this study. The measurements of cell density, pigment composition, astaxanthin content, and total fatty acid content of *H. pluvialis* were evaluated according to the methods used in the previous studies [59–61].

### microRNA profiling of HpEVs

Small RNA high-throughput sequencing technology has revealed compelling details about the miRNA in EVs [51]. In this study, RNA was isolated from HpEVs (*n* = 3) using the miRVana miRNA Isolation Kit (Thermo Fisher Scientific, USA) and the Plant RNA Isolation Aid (Thermo Fisher Scientific, USA) according to the manufacturer’s instructions. Reverse transcription was performed using 125 ng of RNA, quantified using the NanoDrop 2000c, using the miScript Plant RT Kit (Qiagen, Netherlands). microRNA (miRNA) libraries of the HpEVs were constructed using the TruSeq Small RNA Sample Preparation Kit (Illumina, USA) and were sequenced using the Illumina HiSeq2000/2500 System (LC Sciences, USA). The obtained sequences were subjected to stringent filtering using the Fastx-Toolkit (http://hannonlab.cshl.edu/fastx_toolkit/) to remove low-quality reads, repeat sequences, and adaptor sequences, and clean data were generated. The clean data were mapped to the reference genome of *H. pluvialis* [70] by using Bowtie (http://bowtie-bio.sourceforge.net/index.shtml). A BLAST analysis was performed on the mapped reads to detect miRNAs using the miRBase 22.0 database (http://www.mirbase.org/), and the matching miRNAs were considered as conserved miRNAs. The number of reads per miRNA was normalized to transcripts per million reads (TPM). Distribution of miRNA species and expression profiles across six HpEV samples were revealed.

Two miRNA target prediction software, psRobot (http://omicslab.genetics.ac.cn/psRobot/index.php) and TargetFinder, were employed to predict target genes for the identified miRNAs, and identification through at least one software was used as the criteria for selecting candidate target genes. Gene ontology (GO) and Clusters of Orthologous Groups (COG) analyses were performed for functional annotation and classification of these target genes. Because miRNA and target genes are linked, the functional study of miRNA is performed by analyzing target genes. The predicted functions of HpEVs in *H. pluvialis* cells were made based on the interactions of mRNA–miRNA. A hypergeometric test was applied to identify the functions or pathways that were substantially enriched in the significantly differentially expressed miRNA–mRNA pairs compared with all miRNA–mRNA pairs using DESeq2. The aforementioned bioinformatics analyses of the identified miRNA and target genes were performed using the integrated cloud platform of I-Sanger (https://www.i-sanger.com/).

### Quantification the functions of HpEVs

To study the effects of HpEVs and GW4869 on the transcriptome level of *H. pluvialis*, the expression profiles of 17 identified genes, including five carotenoid biosynthesis genes, four fatty acid biosynthesis genes, five cell wall construction-related genes, three cell cycle controlling genes, and the internal control gene (β-actin), were investigated using the real-time fluorescence quantitative PCR (qRT-PCR) method. First, RNA was isolated from HpEV-treated or GW4869-treated *H. pluvialis* cells using the RNA Fast 200 Kit (Fastagen, China) according to the manufacturer’s protocols. Subsequently, the first-strand complementary DNA (cDNA) synthesis was performed using a PrimeScript^TM^RT Reagent Kit with gDNA Eraser (Perfect Real Time) (TaKaRa, Japan). Oligo dT primers were used to synthesize cDNA from 0.5 µg RNA in a 20 µL reaction volume; the synthesized cDNA was used for further qRT-PCR analysis. Finally, qRT-PCR was performed using gene-specific primers listed in Additional file 1: Table S6; the primers were designed using GenBank data, and as reported by Gao et al. [59], Lei et al. [60], Hu et al. [61], and Ma et al. [71]. Expression levels were normalized by comparing with that of the β-actin transcript level, as reported in the previous studies. The qRT-PCR was performed using three independent biological and three technical replicates on an ABI QuantStudio^™^ 6 Flex System (Applied Biosystems, USA) by following a previously described protocol [60, 61, 71, 72] and using an SYBR Green-based PCR assay. The LinRegPCR program was employed to determine the PCR efficiency for each sample, and primer efficiency was calculated by the mean of efficiency values obtained from the individual samples [72]. Expression levels of tested genes were determined and calculated using the 2^–ΔΔCt^ method [73].

### Statistical analysis

The experiments were conducted with biological triplicate from separate microalgal cultures except for miRNA profiling, which was performed in duplicates. In this study, all statistical analyses were performed using the SPSS 19.0 software unless otherwise indicated. In the figures and tables, data are presented as values with standard deviation (mean ± SD). One-way analysis of variance (ANOVA, SPSS 19.0) was performed for statistical analysis, and the *p* values of < 0.05 were considered as statistically significant.

### Supplementary Information

**Additional file 1: Figure S1.** Total miRNA was extracted from HPEVs, and Agilent 2100 detection graphs were measured. **a** Agilent 2100 detection graphs of HPEVs-1 miRNAs. **b** Agilent 2100 detection graphs of HPEVs-2 miRNAs. **c** Agilent 2100 detection graphs of HPEVs-3 miRNAs. **Table S1.** Small RNA sequencing and mapping results. **Table S2.** The de novo transcriptome assembly of small RNA in each libraries. **Table S3.** Classification of HPEVs miRNA precursors in each libraries identified in the present study. **Table S5.** Differentially expressed miRNAs between different stages of HPEVs. **Table S6.** Primers for real time RT-PCR in this study.

**Additional file 2: Table S4.** Full list of the known miRNAs novel miRNA of HPEVs.

## Data Availability

The data that support the findings of this study are available from the corresponding author upon reasonable request.
