# Supplementary material for: Extracellular vesicles involved in growth regulation and metabolic modulation in Haematococcus pluvialis
Source: Biotechnol Biofuels Bioprod. 2024 Jan 28;17:15. doi: 10.1186/s13068-024-02462-z (PMC10823724; doi:10.1186/s13068-024-02462-z)
Supplement: Supplementary file 1 — Additional file 1: Figure S1. Total miRNA was extracted from HPEVs, and Agilent 2100 detection graphs were measured. a Agilent 2100 detection graphs of HPEVs-1 miRNAs. b Agilent 2100 detection graphs of HPEVs-2 miRNAs. c Agilent 2100 detection graphs of HPEVs-3 miRNAs. Table S1. Small RNA sequencing and mapping results. Table S2. The de novo transcriptome assembly of small RNA in each libraries. Table S3. Classification of HPEVs miRNA precursors in each libraries identified in the present study. Table S5. Differentially expressed miRNAs between different stages of HPEVs. Table S6. Primers for real time RT-PCR in this study. [file 13068_2024_2462_MOESM1_ESM.docx]

**Supplementary Information**

Extracellular vesicles involved in growth regulation and metabolite synthesis in *Haematococcus pluvialis*

Qunju Hu^1,2^, Zhangli Hu^1^, Xiaojun Yan^2^, Jun Lu^3^, Chaogang Wang^1,*^

^1^ Shenzhen Key Laboratory of Marine Bioresource and Eco-Environmental Science, Shenzhen Engineering Laboratory for Marine Algal Biotechnology, Guangdong Provincial Key Laboratory for Plant Epigenetics, College of Life Sciences and Oceanography, Shenzhen University, Shenzhen 518060, China;

^2^ College of Marine Science and Technology, Zhejiang Ocean University, Zhoushan 316022, China.

^3^ Auckland Bioengineering Institute, University of Auckland, Auckland 1142, New Zealand

*Corresponding author: Chaogang Wang

Email: [charlesw@szu.edu.cn](mailto:charlesw@szu.edu.cn)

**This file includes 1 supplemental Figure and 6 supplemental Tables, in which Table S4 was supported as a separate file (Additional file [2])**
**Figure S1** Total miRNA was extracted from HPEVs, and Agilent 2100 detection graphs were measured.

**Table S1** Small RNA sequencing and mapping results

**Table S2** The de novo transcriptome assembly of small RNA in each libraries

**Table S3** Classification of HPEVs miRNA precursors in each libraries identified in the present study

**Table S4** Full list of the known miRNAs novel miRNA of HPEVs (**XLSX**)

**Table S5** Differentially expressed miRNAs between different stages of HPEVs

**Table S6** Primers for real time RT-PCR in this study


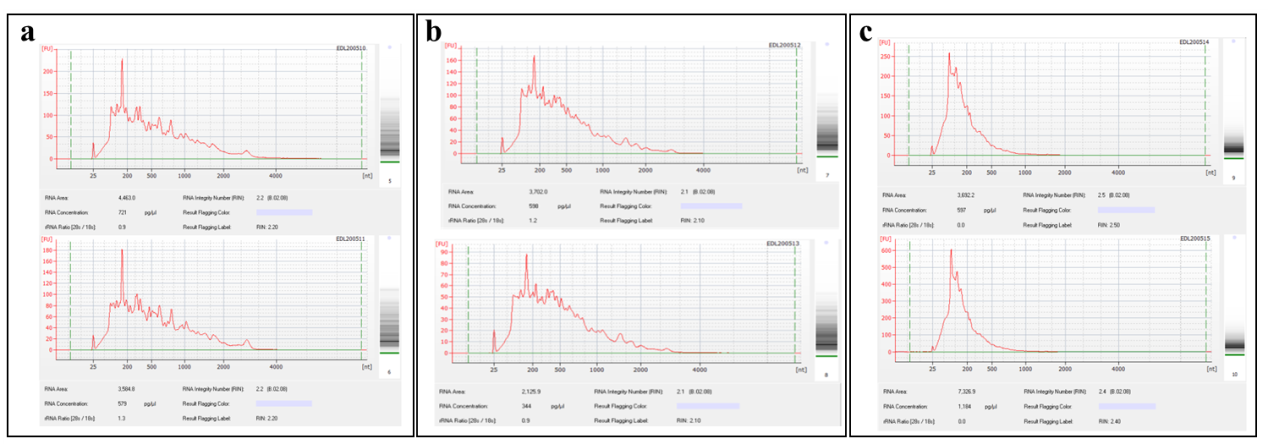


Figure S1 | Total miRNA was extracted from HPEVs, and Agilent 2100 detection graphs were measured. a) Agilent 2100 detection graphs of HPEVs-1 miRNAs. b) Agilent 2100 detection graphs of HPEVs-2 miRNAs. c) Agilent 2100 detection graphs of HPEVs-3 miRNAs

**Table S1 Small RNA sequencing and mapping results.**

| Sample | Clean reads | Clean bases | Error rate(%) | Q20(%) | Q30(%) | GC content(%) | Useful reads(18nt-32nt) |
| --- | --- | --- | --- | --- | --- | --- | --- |
| HPEVs-1-1 | 13,293,511 | 324,983,157 | 0.0251 | 97.85 | 94.17 | 48.37 | 6,089,038 |
| HPEVs-1-2 | 12,088,567 | 282,645,674 | 0.0244 | 98.13 | 94.92 | 47.9 | 5,599,219 |
| HPEVs-2-1 | 11,393,725 | 28,0231,903 | 0.0246 | 98.09 | 94.62 | 50.01 | 6,139,819 |
| HPEVs-2-2 | 11,608,426 | 283,004,426 | 0.0248 | 97.99 | 94.43 | 49.84 | 6,331,481 |
| HPEVs-3-1 | 11,696,454 | 291,388,335 | 0.0243 | 98.26 | 94.91 | 52.34 | 6,814,363 |
| HPEVs-3-2 | 13,115,458 | 340,502,457 | 0.0244 | 98.2 | 94.75 | 52.22 | 8,157,045 |

**Table S2 The de novo transcriptome assembly of small RNA in each libraries.**

| Types\Samples | HPEVs-1-1 | HPEVs-1-2 | HPEVs-2-1 | HPEVs-2-2 | HPEVs-3-1 | HPEVs-3-2 |
| --- | --- | --- | --- | --- | --- | --- |
| known miRNA | 53,827 | 40,669 | 36,489 | 38,462 | 19,009 | 17,289 |
| novel miRNA | 1080 | 831 | 349 | 629 | 538 | 597 |
| rRNA | 1,788,970 | 1,927,113 | 3,160,683 | 3,095,962 | 3,733,111 | 4,738,122 |
| tRNA | 24,293 | 13,255 | 17,813 | 21,096 | 19,650 | 18,725 |
| snoRNA | 1,405 | 697 | 2,335 | 2,510 | 8,359 | 9,954 |
| snRNA | 1,835 | 1,602 | 2,958 | 2,886 | 3,482 | 4,154 |
| repbase | 50,094 | 18,997 | 16,259 | 19,243 | 15,830 | 14,794 |
| exon | 3,110 | 2,321 | 2,958 | 3,395 | 5,101 | 6,230 |
| intron | 50,757 | 33,248 | 34,124 | 30,301 | 26,887 | 31,595 |
| unknown | 4,113,667 | 3,560,486 | 2,865,851 | 3,116,997 | 2,982,396 | 3,315,585 |
| total | 6,089,038 | 5,599,219 | 6,139,819 | 6,331,481 | 6,814,363 | 8,157,045 |

**Table S3 Classification of HPEVs miRNA precursors in each libraries identified in the present study.**

| Sample | known miRNAs | novel miRNAs | total |
| --- | --- | --- | --- |
| HPEVs-1-1 | 16 | 33 | 49 |
| HPEVs-1-2 | 17 | 23 | 40 |
| HPEVs-2-1 | 39 | 13 | 52 |
| HPEVs-2-2 | 18 | 21 | 39 |
| HPEVs-3-1 | 39 | 15 | 54 |
| HPEVs-3-2 | 20 | 18 | 38 |
| Total | 93 | 70 | 163 |

**Table S5 Differentially expressed miRNAs between different stages of HPEVs.**

| miRNA\Group | HPEVs-3 vs. HPEVs-1 |
| --- | --- |
| Nov-m0043-3p | up |
| Nov-m0029-5p | up |
| Nov-m0039-5p | up |
| atr-miR8590 | down |
| Nov-m0049-3p | down |
| Nov-m0068-5p | down |
| Nov-m0052-3p | down |
| Nov-m0028-3p | down |
| Nov-m0009-3p | down |
| Nov-m0048-3p | down |
| Nov-m0032-5p | down |
| Nov-m0002-5p | down |

**Table S6 Primers for real time RT-PCR in this study.**

| Primer | Primer Sequence (5’-3’) | References |
| --- | --- | --- |
| *PSY-F* | CGATACCAGACCTTCGACG | Gao *et al*., 2012 |
| *PSY-R* | TGCCTTATAGACCACATCCAT |  |
| *PDS-F* | AAGTTCAGACCCACTCAGCG | Gao *et al*., 2012 |
| *PDS-R* | AGTCCTCAACAATGGCCTCG |  |
| *LYC-F* | TGGAGCTGCTGCTGTCCCT | Gao *et al*., 2012 |
| *LYC-R* | GAAGAAGAGCGTGATGCCGA |  |
| *CRTR-B-F* | ACACCTCGCACTGGACCCT | Gao *et al*., 2012 |
| *CRTR-B-R* | GTATAGCGTGATGCCCAGCC |  |
| *BKT-F* | CAATCTTGTCAGCATTCCGC | Gao *et al*., 2012 |
| *BKT-R* | CAGGAAGCTCATCACATCAGAT |  |
| *BC-F* | CAAGAAGGTGATGATCGCCA | Lei *et al*., 2012 |
| *BC-R* | GACGTGCAGCGAGTTCTTGTC |  |
| *ACP-F* | CAGCTCGGCACTGACCTTG | Lei *et al*., 2012 |
| *ACP-R* | CAAGGGTCAGCTCGAACTTCTC |  |
| *SAD-F* | CCGAGCCCAAGCTTCTAGTG | Lei *et al*., 2012 |
| *SAD-R* | TTTGCCTCCATGTAATCCCC |  |
| *FAD-F* | GTAGGTCACCACGTCCAGCC | Lei *et al*., 2012 |
| *FAD-R* | CTTGATAGGCATGCTGGGTGT |  |
| *CS-F* | TCCGATCCGACCCACCACACT | Guo *et al*., 2021 |
| *CS-R* | AGTGAAGGCCCACAGGCAACC |  |
| *OGH-F* | GTACACCAGCGCGGAATGCGA | Guo *et al*., 2021 |
| *OGH-R* | GGGCACATGGTTATGGCGCAGT |  |
| *MND-F* | GTCACCGCAGACGGCACTGT | Guo *et al*., 2021 |
| *MND-R* | CCGCTGCCGTCCAGGTATCG |  |
| *MTD-F* | GCCGCAAAAGCTGTCCCT | Guo *et al*., 2021 |
| *MTD-R* | CCACCACGCCCACTTTCA |  |
| *GPE-F* | CAGGCCAGATGCCGTGTTT | Guo *et al*., 2021 |
| *GPE-R* | GGGCAAAGCCATGCTGCT |  |
| *CYCA-F* | TAAGCCAGAGCAGCAGGAAC | This study |
| *CYCA-R* | GCACTTTCACATGCACCCTG |  |
| *CYCB-F* | TTGCGGGCAAGGAAGTTGTA | This study |
| *CYCB-R* | CCTTGAGCAGAAGCCAGTCA |  |
| *CDC45-F* | TGGTCAGCCAGTATGCAGTG | This study |
| *CDC45-R* | CTAGTTCCATGCATGTGCGC |  |
| *β-Actin-F* | ACCTCAGCGTTCAGCCTTGT | Gao *et al*., 2012 |
| *β-Actin-R* | TGGTCCACGACACCATCAAC |  |
